# Rate of hepatitis C viral clearance by human livers in human patients: Liver transplantation modeling primary infection and implications for studying entry inhibition

**DOI:** 10.1371/journal.pone.0180719

**Published:** 2017-07-21

**Authors:** Michael G. Hughes, William W. Tucker, Sreelatha Reddy, Michael E. Brier, David Koch, Craig J. McClain, Colleen B. Jonsson, Nobuyuki Matoba, Donghoon Chung

**Affiliations:** 1 Department of Surgery, University of Louisville, Louisville, Kentucky, United States of America; 2 Department of Medicine, University of Louisville, Louisville, Kentucky, United States of America; 3 Department of Medicine, Medical University of South Carolina, Charleston, South Carolina, United States of America; 4 Department of Pharmacology and Toxicology, University of Louisville, Louisville, KY, United States of America; 5 Robley Rex Louisville Veteran Affairs Medical Center, Louisville, Kentucky, United States of America; 6 Department of Microbiology, University of Tennessee, Knoxville, Tennessee, United States of America; 7 Department of Microbiology and Immunology, University of Louisville, Louisville, Kentucky, United States of America; Centre de Recherche en Cancerologie de Lyon, FRANCE

## Abstract

To better understand the dynamics of early hepatitis C virus (HCV) infection, we determined how rapidly non-cirrhotic HCV-uninfected liver allografts clear HCV from the circulation of cirrhotic HCV-infected patients at the time of transplantation but before administration of immunosuppression. Specifically, we characterized serum HCV kinetics during the first 90 min of reperfusion for 19 chronically HCV-infected patients transplanted with an HCV-uninfected liver by measuring serum viral load immediately prior to reperfusion (t = 0) and then every 15 min for a total of 90 min (t = 90). Immunosuppression was withheld until all samples were taken to better model primary infection. During this period, rates of viral clearance varied more than 20-fold with a median rate constant of 0.0357 1/min, range 0.0089–0.2169; half-life (minutes) median 19.4, range 3.2–77.8. The majority of viral clearance occurred within the first 60 min. The amount of blood transfused during this 90-min period (a potential confounding variable of this human liver transplant model of primary infection) accounted for 53% and 59% of k (r = 0.53, p = 0.05) and half-life (r = 0.59, p = 0.03) variability, respectively. No other clinical variables tested (age, allograft weight, and degree of reperfusion injury as assessed by peak postoperative ALT or AST) accounted for the remaining variability (p>0.05). ***Conclusion*:** In a human liver transplant model of primary infection, HCV rapidly clears the bloodstream. With approximately 90% of clearance occurring in the first 90 minutes of reperfusion, studies of HCV entry inhibition could utilize rate of clearance during this early period as an outcome measure.

## Introduction

The study of HCV pathogenesis is complicated by the lack of relevant animal models. Chimpanzee, murine, and a murine model with hepatocytes of human origin susceptible to HCV infection have been developed; unfortunately, all have limitations. Despite established HCV infection in individuals undergoing liver transplantation for HCV, the previously uninfected allograft is not infected until after reperfusion. Therefore, transplant represents a primary infection of that organ. Liver transplantation, therefore, represents the only model to ethically and prospectively study infection of a human liver in humans.

Viral kinetics, or change in circulating viral loads over time, has been used for various purposes. Very high viral loads at the time of transplant have been shown to predict inferior outcomes [1]. This is likely due to a greater viral inoculum, leading to a greater burden of infection and accelerated disease progression analogous to primary infection [2–4]. The observation that viral load within the first month after transplant predicts outcomes more accurately than later time points [5, 6] suggests that early viral pathogenesis may have a critical role in subsequent liver injury. Garcia-Retortillo et al. [7] determined that serum viral levels decrease with reperfusion, and that viral clearance occurred more slowly during reperfusion (first sample taken 4 hours after reperfusion) than during the anhepatic phase. We hypothesized that these contradictory findings were due to missing the initial viral clearance (earlier than 4 hours). The purpose of the current study was to more fully characterize initial HCV clearance by livers by determining the viral kinetics immediately upon allograft reperfusion (first 90 min). To more accurately model primary infection, immunosuppression (including steroids) was withheld until all samples were collected. This was a key difference from other published studies on HCV kinetics. The current study significantly adds to the field by fully characterizing the variability of initial viral clearance in the absence of immunosuppression.

Better understanding the kinetics of viral clearance during this time period can help inform study design around entry inhibition. Though great strides have been made recently in HCV treatment with a steady stream of direct acting antivirals (DAAs) entering the market, there may remain a role for entry inhibition[8–10]. If the initial rate of viral clearance were to be further characterized, then studies of HCV entry inhibitors could use these data as a short-term outcome measure.

## Materials and methods

### Human subjects

This study conformed to the ethical guidelines of the 1975 Declaration of Helsinki and was approved by the Human Investigation Committee at the Medical University of South Carolina (Institutional Review Board protocol HR# 18687). All patients with end-stage liver disease secondary to HCV injury undergoing liver transplantation with an HCV-uninfected allograft were offered enrollment in the study. Patients were excluded if previously transplanted or no longer viremic at time of transplantation. Informed consent was obtained from all patients in writing prior to transplantation. No donor organs were obtained from executed prisoners or other institutionalized persons.

### Patient samples

Serum samples were drawn immediately prior to allograft reperfusion and then every 15 min for 90 min. Blood samples were processed within 4 hours of collection and serum was stored at -80^o^ C. All blood products that were administered during surgery underwent nucleic acid testing for HCV prior to transfusion. Immunosuppression (including steroids) was withheld until all samples were collected. Samples were not collected after 90 min, as results would have been confounded by immunosuppression. Allografts were weighed prior to implantation to determine impact on rates of viral clearance.

### Standards

The AcroMetrix HCV-S Panel (Life Technologies, Frederick, MD) was used as the World Health Organization (WHO) standard. All viral load determinations were made relative to the WHO standard through construction of a standard curve for absolute quantitation of HCV in International Units (IU)/mL. Use of the WHO standard allows for comparison across samples, patients, and institutions.

### Internal control

As viral load measurements and kinetic calculations were to be compared across a short time period with small differences considered potentially significant, Armored HIV RNA Quant (Asuragen Inc., Austin, TX) was added to each serum sample as an exogenous internal control to account for process variability. HIV was selected as the internal control because all patients in the study were HIV negative and the control was commercially available. Armored RNA HIV Quant contains a synthetic RNA sequence encapsulated within a bacteriophage coat protein to stabilize the RNA and protect it from nuclease degradation; hence, this RNA control can be extracted along with the target HCV RNA and standards. Extraction variability from experiment to experiment was monitored by quantitative real time PCR.

### RNA extraction

Total HCV RNA and internal controls were extracted from samples and standards using the QIAamp UltraSens Virus Kit (Qiagen, Germantown, MD), according to the manufacturer’s instructions with minor modifications. RNA was extracted from 990 μl of samples or standards spiked with 10 μl of Armored HIV RNA Quant (5,000 copies) as an internal positive control. RNA was eluted at a consistent volume, stored in small aliquots at -80^°^C, and subjected to no more than one freeze-thaw cycle.

### Primers and probes

Full length HCV genomic sequences were downloaded from the Los Alamos National Laboratory [11]. These sequences were aligned and primers were designed with CLC Main Workbench 5 (CLC Bio, Cambridge, MA) to bind only 100% conserved regions across all genotypes within the 5’-untranslated region (5’UTR). Each primer and probe set was BLAST-searched across the human genome, and those with cross-homology were discarded from future analysis. Selected primers and probes for HCV and control were determined to have no cross-homology.

As conserved regions were too short for conventional primer and probe design, primers and probes were modified with Locked Nucleic Acids (LNA) (Exiqon, Woburn, MA) to increase melting temperature (Tm) and limit secondary structure. LNA-substituted primers were obtained from Exiqon (+ precedes the LNA substituted base): HCV forward binding primer 5’-C+CGGGAGAGCCA-3’, HCV reverse binding primer 5’-T+CCA+AGAAAGGACCC-3’, control forward binding primer 5’-G+AA+GC+TGCAGAATG-3’, and control reverse binding primer 5’-T+CC+TG+CTATGTCAC-3’. LNA-substituted hydrolysis probes were obtained from Integrated DNA Technologies (IDT, Coralville, Iowa): HCV forward binding probe 5’/6-FAM/T+GCGG+AACCGGT+GAG/IABlkFQ/3’ and control forward binding probe 5’/CY5/G+ATG+AGA+GAA+CCA+AGG/IABlk RQ/3’. Control primers and probe targeted the gag region of HIV.

### Quantitative real-time RT-PCR

Reverse transcription, amplification, and quantitation of HCV and control RNA were simultaneously performed as a 1-step reaction with QuantiTect Virus (Qiagen), according to the manufacturer’s instructions. For consistency, a primer-probe master mix (20x) containing HCV and control primers and probes was diluted into TE buffer (10 mM Tris with 0.1 mM EDTA) to achieve final concentrations of 400 nM for primers and 250 nM for probes. Reactions were conducted in triplicate at 50^o^ C for 30 min, 95^o^ C for 5 min, and then 45 cycles of 95^o^ C for 15 sec and 60^°^C for 45 sec (Applied Biosystems 7500 real-time PCR system).

Validation studies of the assay determined that the standard curve was linear (y = -3.359x + 42.440; r^2^ = 0.99749) with a dynamic range of 1.5x10^2^–2.5x10^7^ IU/mL. Limit of Detection (LOD) (n = 21) was 75.5 IU/mL. Values below the LOD were reported as none detected (N/D). Limit of Quantitation (LOQ) (n = 7) was 155.5 IU/mL. The intra-assay Coefficient of Variation (CV) was 0.20–2.60% for HCV and 0.51–2.24% for HIV. The inter-assay Coefficient of Variation (CV) was 1.65–4.40% for HCV and 1.43–3.01% for HIV.

### Analyses

For each sample, HCV levels were normalized to controls by subtracting control threshold cycle (CT) from HCV CT. The absolute value for the unknown HCV samples was calculated from the standard curve using linear regression and normalized CT. Amplification efficiencies of HCV and control were similar. Standard curves were constructed for each reaction. Elimination (or HCV clearance) rate constants were calculated using linear regression of the natural log of the concentration vs. time. Half-life was calculated as ln(2/k). Two-tailed Pearson correlation analysis was done using different clinical variables and HCV elimination constants. Statistical analyses were performed with JMP 8.0.2 (SAS Institute Inc., Cary, NC).

## Results

### Clinical characteristics

Of the 19 patients enrolled in the study, 4 were female and 15 were male (Table 1). Three patients were African-American, one was Hispanic, and the remainder was Caucasian. The median patient age was 57 years (range: 48–73 years). Most HCV was genotype 1a (n = 10), followed by genotype 1b (n = 5), genotype 3 (n = 3) and genotype 2b (n = 1). Of those patients transfused during the 90-min reperfusion period, a median of 2 units was administered (range: 1–6 units). Transfusion data for the first four patients were not collected prospectively. As retrospective analysis could not determine when blood was given relative to the 90-min reperfusion phase, these patients were excluded from any analysis regarding transfusion. Following transplantation, 5 patients developed significant ischemia-reperfusion injury with a peak postoperative serum ALT >1,000 (median: 640; range: 134–2,520). Primary non-function developed in one allograft, resulting in patient death. All allografts studied weighed between 1,206 and 3,660 grams (median: 1,510 grams).

**Table 1:** Patient demographics.

Patient Number	Age (yrs)	Gender	Ethnicity	Genotype	Blood Transfused (units)
1	58	Female	Caucasian	1b	n/a
2	52	Male	Caucasian	1a	n/a
3	55	Male	Caucasian	3	n/a
4	57	Male	Hispanic	1a	n/a
5	73	Male	Caucasian	1b	2
6	59	Male	Caucasian	1a	2
7	54	Male	Caucasian	3a	2
8	55	Male	Caucasian	1a	2
9	54	Male	Caucasian	1b	2
10	48	Male	African-American	1a	1
11	58	Male	African-American	1a	0
12	58	Male	African-American	1a	0
13	59	Male	Caucasian	3	1
14	57	Female	Caucasian	1b	6
15	52	Male	Caucasian	1b	1
16	53	Female	Caucasian	1a	0
17	63	Male	Caucasian	2b	0
18	56	Female	Caucasian	1a	0
19	56	Male	Caucasian	1a	0

### Serum viral kinetics during initial allograft reperfusion

**Fig 1:**
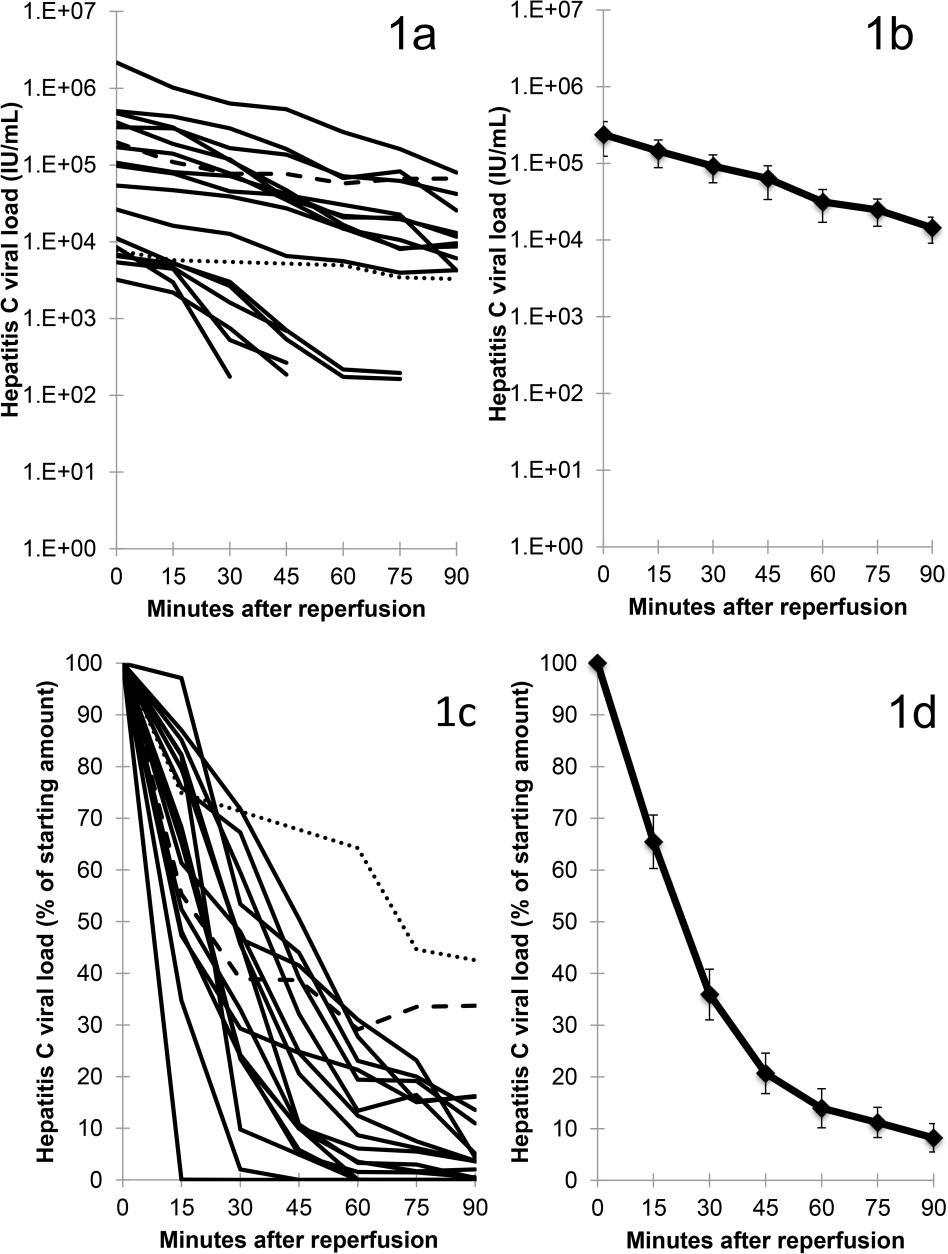
Allografts rapidly remove HCV from patient circulation. **(A)** and **(C)**: Each line represents an individual patient. The dotted (patient 1) and dashed (patient 5) lines represent the patients with the slowest rates of viral clearance. **(B)** and **(D)**: Line represents the average of all patients with error bars representing standard error. **(A)** and **(B)**: Absolute values for HCV are presented. **(C)** and **(D)**: HCV values relative to starting amount are presented. During reperfusion, the rate of viral clearance varied more than 20-fold with a median rate constant of 0.0357 1/minute (range 0.0089–0.2169 1/minute) and median half-life of 19.4 minutes (range 3.2–77.8 minutes).

The viral load immediately prior to reperfusion was quite variable (median: 57,000 IU/mL; range: 1,394–2,157,780 IU/mL) (Table 2). With reperfusion, viral loads decreased rapidly, with most viral clearance within the first 60 min of reperfusion (Fig 1). Of the 14 patients with detectable HCV at 90 min, viral levels reached a nadir as early as 60 min (patient 5) and 75 min (patients 2, 11, and 12). Only patient 5 appeared to have new viral production exceed clearance or alternatively to demonstrate a biphasic decline during the time period studied (r^2^ for the natural log of viral load over time was 0.6918). This can be appreciated in Fig 1, where patient 5 deviates from the log-linear slope. For the others, clearance remained log-linear (r^2^: median 0.9402; range 0.8484–0.9847). For five patients, viral levels went below our LOD more than once during the time period studied, which was likely due to a lower starting viral load (5.1 x 10^3^ ± 1.2 x 10^3^ IU/ml vs. those patients with levels below LOD no more than once: 3.0 x 10^5^ ± 1.4 x 10^5^ IU/mL, p = 0.03).

**Table 2:** HCV clearance during initial allograft reperfusion. All HCV levels were normalized to internal control. Internal control was detectable for all samples. Measures of HCV clearance were: k (rate constant) and t_1/2_ (half-life). (a) r^2^ was determined from the natural log of HCV viral load over time. N/A: sample not available. N/D: none detected.

Patient Number	HCV levels (IU/mL) after reperfusion							HCV uptake		r2 (a)
	0 min	15 min	30 min	45 min	60 min	75 min	90 min	k (1/min)	t1/2 (min)	
1	7,689	5,760	N/A	N/A	4,938	3,430	3,270	0.0089	77.8	0.9194
2	26,301	16,133	12,634	6,525	5,605	3,952	4,266	0.0208	33.3	0.9358
3	1,394	N/D	N/D	N/D	N/D	N/D	N/D	-		-
4	359,140	188,144	118,546	36,662	21,855	19,704	13,058	0.0371	18.7	0.9540
5	197,576	109,334	76,847	76,394	57,554	66,222	66,630	0.0105	65.9	0.6918
6	11,057	5,326	2,653	537	173	163	N/D	0.0593	11.7	0.9720
7	3,202	2,186	751	186	N/D	N/D	N/D	0.1308	5.3	0.8966
8	6,852	4,590	1,620	689	N/D	N/D	N/D	0.1203	5.8	0.9061
9	97,582	77,618	45,507	40,498	30,277	22,589	4,241	0.0291	23.8	0.8404
10	2,157,780	1,020,972	632,272	530,701	268,823	161,162	79,843	0.0342	20.2	0.9847
11	473,687	309,205	114,837	46,996	16,633	8,029	9,609	0.0488	14.2	0.9574
12	54,061	47,061	38,819	27,322	15,006	8,258	8,697	0.0236	29.3	0.9432
13	8,519	2,957	175	N/D	N/D	N/D	N/D	0.2169	3.2	0.9263
14	5,400	4,436	528	266	N/D	N/D	N/D	0.1471	4.7	0.8895
15	6,538	5,207	3,019	703	217	196	N/D	0.0605	11.4	0.9488
16	170,375	140,286	77,737	35,066	14,732	10,535	6,077	0.0395	17.5	0.9812
17	502,699	428,142	299,458	161,617	67,314	82,761	25,607	0.0323	21.4	0.9372
18	310,180	301,204	165,655	136,395	71,508	62,075	42,107	0.0236	29.3	0.9719
19	106,639	81,008	71,715	41,739	20,717	20,414	11,660	0.0251	27.6	0.9628

**Fig 2:**
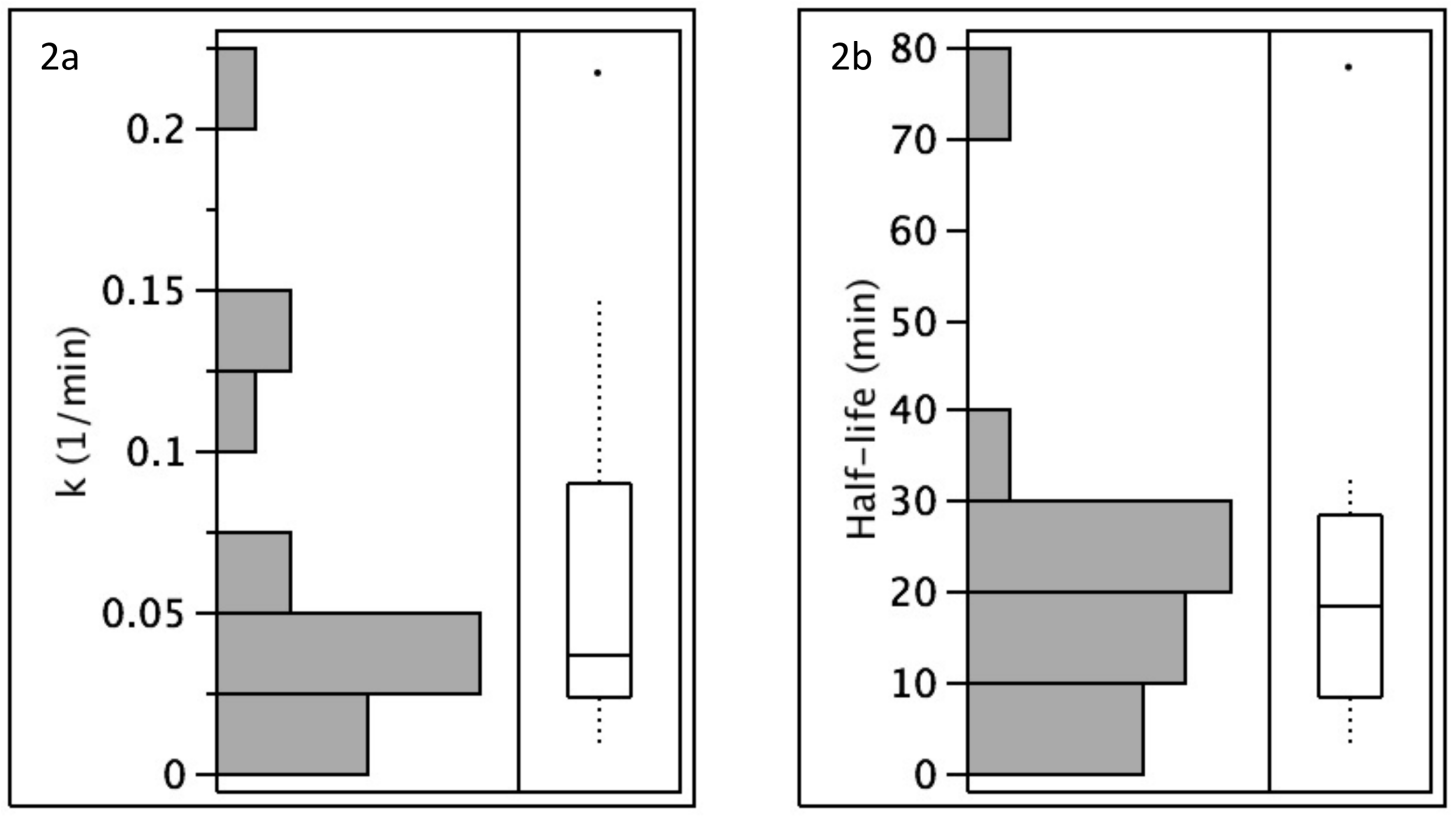
Distribution of rates of HCV clearance by allografts. Viral clearance was expressed as k (1/min) and half-life (min). Histograms **(A**) and box plots **(B**) demonstrate that these distributions are skewed toward slower clearance, although outliers impacted half-life less than k.

HCV viral clearance after graft reperfusion followed first-order elimination kinetics (decrease in HCV concentration was log-linear). During reperfusion, the rate of viral clearance varied more than 20-fold with a median rate constant of 0.0357 1/min (range, 0.0089–0.2169 1/min) and median half-life of 19.4 min (range, 3.2–77.8 min) (Table 2). The rate of viral clearance appeared greatest for genotype 3 (median rate constant of 0.1739, range 0.1308–0.2169), followed by much lower rates that were similar between genotypes 1a (median 0.0357, range 0.0208 - .1203), 1b (median 0.0291, range 0.0089–0.1471), and 2b (single patient 0.0323). Of the four outliers (patients 7, 8, 13, 14) with more rapid clearance (Fig 2), two were genotype 3 (patients 7 and 13). The third genotype 3 patient (patient 3) could not be assessed for rate of clearance as he cleared virus within 30 minutes (Table 2). With so few genotype 3 patients, we could not determine if these differences were statistically significant. Patient 14 was transfused 6 units of blood. As this was 4 units more than any other patient, this high rate of clearance could be accounted for HCV-infected blood loss replaced with HCV-uninfected blood. Only patient 8 (genotype 1a) had no apparent explanation for his outlier status.

### Clinical predictors of serum viral kinetics

**Fig 3:**
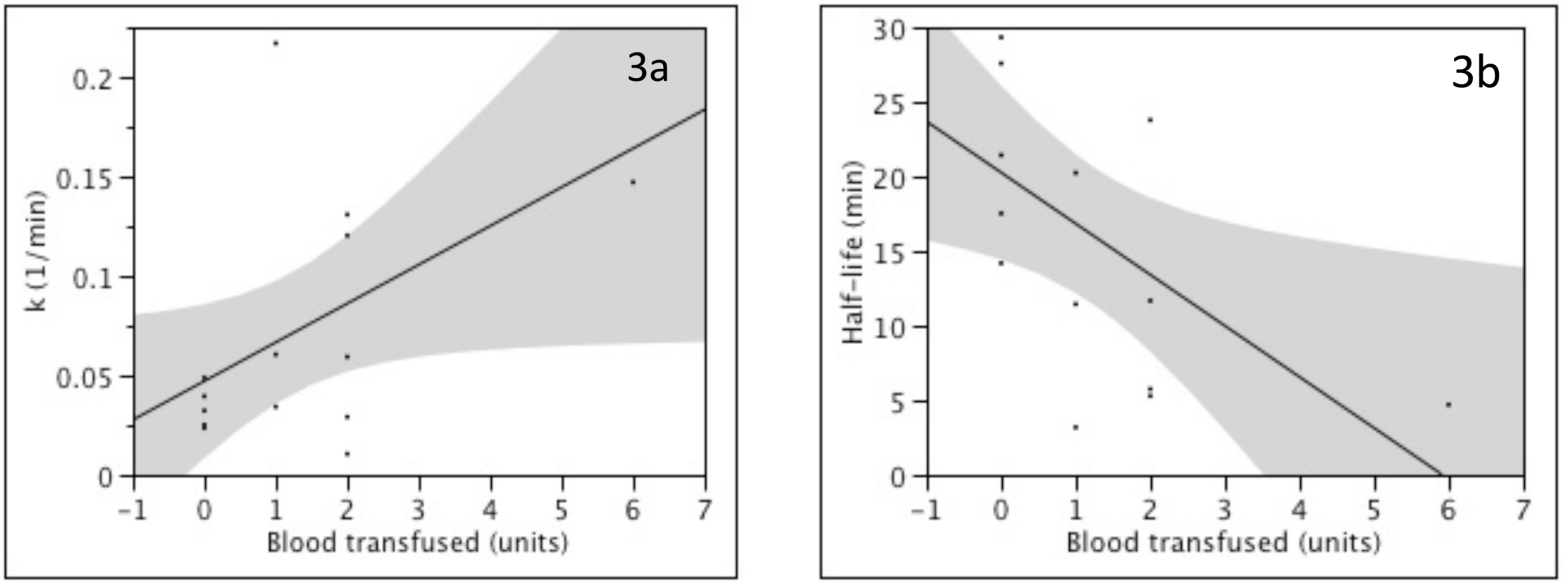
Transfusion of blood during reperfusion impacted HCV kinetics. Increasing volumes of blood transfused during the reperfusion period increased k (**A**: r = 0.53, p = 0.05) and shortened half-life (**B**: r = 0.59, p = 0.03) (n = 14).

The rate of viral clearance (or decrease in circulating levels) increased with greater volumes of blood transfused during the 90-min reperfusion period (Fig 3). This provides further evidence that blood transfusion could explain the outlier status for patient 14. Those patients transfused 1 or 2 units of blood had an approximately two-fold faster clearance and shorter half-life (median k = 0.0599, range 0.0291–0.217; median half-life = 11.6 min; range 3.2–23.8 min) than those not transfused (median k = 0.0287, range 0.0236–0.0488; median half-life = 24.5 min; range 14.2–29.3 min). No significant correlations were found for age (r = 0.08, p = 0.8), allograft weight (r = 0.04, p = 0.9), or reperfusion injury as assessed by peak post-operative ALT (r = 0.3, p = 0.2) and peak post-operative AST (r = 0.3, p = 0.2). Patient 5 was excluded from these analysis as his rate of HCV decline was not log-linear.

### Post-reperfusion serum viral kinetics

**Fig 4:**
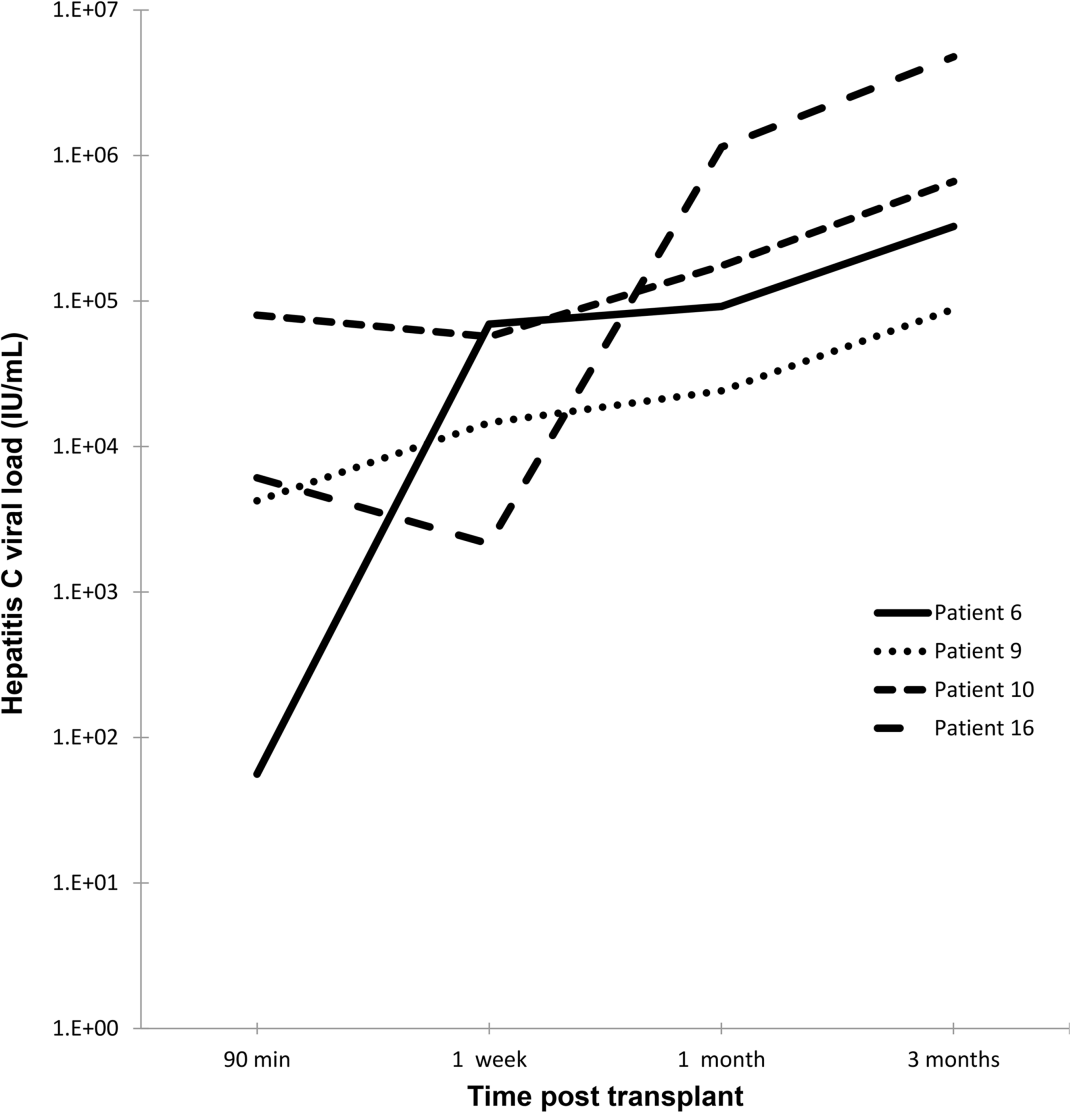
Examples of HCV recurrence in patient serum after liver transplantation. Each line represents an individual patient’s serum HCV viral load over the 3 months following transplantation.

To determine how well our findings correlated with other studies, we determined vial levels at 90 min, 1 week, 1 month and 3 months for patients with a complete set of samples (n = 4). Viral load increased from 90 min to 1 week for patients 6 and 9 and decreased for patients 10 and 16 (Fig 4). For all 4 patients, viral load increased from 1 week to 1 month to 3 months. There was much more variability seen after 90 min than before 90 min.

## Discussion

The purpose of the current study was to better understand how rapidly hepatitis C virus (HCV) clears the bloodstream. As the only ethical way to infect a human liver in a human patient is by transplanting an HCV-uninfected liver into an HCV-infected recipient, we calculated the rates of viral clearance from the bloodstream during liver transplant as a surrogate for viral entry. To more accurately model primary infection, immunosuppression was withheld during the study period. This is a key difference from other published studies on HCV kinetics during liver transplantation.

We hypothesized that the rate of viral clearance (and likely entry) varies widely during initial infection and focused our investigation on the first 90 min of reperfusion to ensure that the dynamics of initial infection were captured. Decreasing serum viral levels over the 90 min of initial reperfusion were considered to represent viral clearance by allografts, as it has been previously demonstrated that serum viral levels dramatically decrease with transition from the anhepatic phase to the reperfusion phase of liver transplant [7, 12]. The current study significantly adds to existing knowledge regarding this transition by 1) evaluating earlier time points and 2) excluding immunosuppression as a confounding variable.

We elected to use an in-house HCV qRT-PCR assay rather than utilize a commercial assay to minimize variability. The internal controls for the commercial assays were qualitative rather than quantitative and therefore could not be used for calibration. For our study, we anticipated that we would need to detect potentially very small changes in viral load as we were going to sample every 15 minutes. We therefore used a quantitative internal control (Armored HIV RNA quant) that could be used to calibrate our HCV levels and reduce variability. Compared with commercially available assays, our variability was lower [13], though we did sacrifice some sensitivity. For this study, we prioritized minimizing variability over maximizing sensitivity.

In our current study of 19 patients who were viremic at the time of reperfusion, the rate of viral clearance by allografts varied more than 20-fold but demonstrated first order kinetics for all patients but one (patient 5). The linearity of the process throughout the 90 min of the study suggests that virus may be cleared at a rate greater than production. Most viral clearance occurred in the first 60 min of reperfusion. Of the tested clinical variables, only blood transfusion could account for any of the observed variability. Patient 5 deviated from log-linear towards the end of the 90-minute period. Though this could be due the newly infected liver supporting rapid viral replication and release back into the bloodstream, this plateau could rather represent the intermediate phase of a biphasic decline consistent with what has been described in other studies [7, 14]. If the minimal intra-cellular delay time is truly 6 hours [14], then this more likely represents a second, extra-hepatic replication compartment that generated a more significant contribution to HCV levels for patient 5 than for the other patients [14]. The duration of our study was too short to calculate the contribution of a second compartment for these patients.

Bleeding and resulting blood transfusion is a likely a confounding variable for this model of primary infection. Virus is lost during bleeding with a reduction in circulating blood volume. Restoring blood volume with transfusion of HCV-uninfected blood and fluids then dilutes out circulating viral levels. However, blood transfusion could not fully account for the observed differences in this study. The amount of blood transfused during this 90-min period accounted for 53% and 59% of k (r = 0.53, p = 0.05) and half-life (r = 0.59, p = 0.03) variability, respectively. It may be that blood transfusion is an imperfect marker for blood loss and volume replacement. Patients transfused just 1 or 2 units of blood were observed to clear virus twice as quickly as those that did not. It seems unlikely that a <500 cc/unit blood transfusion could dilute viral levels to this degree when circulating blood volumes are typically 3–5 liters. Measuring actual blood loss in real-time during reperfusion was not possible as this process is too dynamic. We therefore propose that the intrinsic rate viral clearance would be best estimated by the median rate of clearance for those patients not transfused (0.0287 1/min). However, this still likely overestimates the true rate of viral clearance as it cannot separate out the contribution of degradation or other non-hepatic clearance mechanisms. If we had determined the rate of clearance during the anhepatic phase, then we could have used these data to more accurately determine hepatic clearance. This represents a limitation of our study design.

The study was designed to carefully and prospectively analyze a very discrete period during the transplant event and follow up on the findings of Garcia-Retortillo et al. [7] and Powers et al. [12]. In a study of 20 patients undergoing liver transplantation, Garcia-Retortillo et al. showed that virus decreased with reperfusion despite having a longer half-life (3.4 hours compared with 2.2 hours during the anhepatic phase). This discrepancy is likely because they did not measure any circulating viral levels during the first 4 hours of reperfusion. By evaluating the first 90 min of reperfusion in 15-min intervals, the current study shows that clearance occurs much earlier and faster (t_1/2_ 19.4 min). The half-life of HCV during reperfusion (as calculated by Garcia-Retortillo et al.) was likely longer than the anhepatic phase because viral production by the newly infected allograft was starting to increase relative to clearance.

In a smaller study of 6 patients, Powers et al. (9) sampled serum during reperfusion earlier than Garcia-Retortillo et al. (10). However, the Powers’ study still did not capture the first hours of reperfusion, missing most clearance, and calculated viral clearance by lumping the anhepatic phase within the first 4 hours of reperfusion. The purpose and design of their study was not to evaluate viral clearance, but rather to characterize viral resurgence following initial reperfusion. They calculated a similar half-life during reperfusion (3.4 hours) as Garcia-Retortillo et al., likely because they also started 4 hours after reperfusion. Their graphical depiction of viral levels does show, like Garcia-Retortillo et al., that the largest decrease in viral levels occurred between the last anhepatic and the first reperfusion samples drawn. Therefore, viral clearance during initial reperfusion (as described in the current study) links viral decay during the anhepatic phase and viral resurgence during later reperfusion (as described by these two prior studies). We have included the viral kinetics following the 90-minute reperfusion phase from 4 patients to show that our findings are similar to the other cited studies when measured over the same time period.

It appears that the clearance mechanism was not saturated in any of the patients studied. This was determined by the observation that the concentrations fall in a linear fashion (on the straight line of natural log of HCV concentration over time). It is therefore unlikely that cell surface receptors for the virus (such as CD81 [15], SR-BI [16], Claudin-1 [17] and Occludin [18]) were present at low enough levels to become saturated. With such high titer inoculum relative to other primary infections (e.g. needle stick transmission), it is unlikely other modes of transmission would result in receptor saturation either. During the first 90 min of reperfusion, new viral production was minimal relative to clearance due to the HCV concentration over time not departing from linearity. Therefore, studies of viral evolution during this time period are unlikely to be significantly confounded by production of new mutations [19, 20].

The large variation in rates of viral clearance not fully accounted for by the tested clinical variables must be explained by other factors. As clearance is determined by blood flow and extraction efficiency, variable perfusion pressure during reperfusion will contribute to the observed differences. This is a likely contributor as hemodynamic instability occurs frequently during reperfusion. Furthermore, differences in extraction efficiency could be due to differences in allograft or viral factors. Allografts may differ in HCV receptor density and/or turnover [20, 21] and therefore may vary in susceptibility to infection. In a study of liver transplant recipients in the first year following transplantation, Mensa et al. found that higher concentrations of SR-BI in the allograft correlated with more rapid clearance of HCV in the early reperfusion phase (first 24 hours), whereas higher levels of occludin and claudin-1 correlated with faster rate of viral production in the following week [22]. Alternatively, a study targeting SR-BI showed that an SR-BI antagonist, ITX5061, had no impact on rates of viral clearance in the first 24 hours, but may have limited subsequent viral rebound [23]. Additionally, viral populations have been shown to differ between patients, leading to variable levels of infectivity [19, 20, 24]. The same study of ITX5061 showed that SR-BI blockade limited viral quasispecies evolution at hypervariable region 1 of E2 [23], further supporting that SR-BI may bind HCV at HVR1 [25]. Patients therefore may differ in viral inoculum fitness.

We believe that the data presented herein can provide background to design clinical studies measuring the efficacy of HCV entry inhibitors. Entry inhibitors can play a role during liver transplant by preventing transmission of recipient bloodstream derived HCV into the newly transplanted uninfected liver without damaging vulnerable hepatocytes [8, 10]. Given that secondary non-hepatic compartments for viral production may exist, such inhibitors may need to address these compartments through anti-viral activity, either through an intrinsic mechanism or when given in combination with direct acting anti-viral agents [23]. Rates of viral clearance over the first 90 minutes of reperfusion would be relevant outcome measure for entry inhibitors that target either the virus or host-entry factors. Liver transplantation could be used to study the impact of these inhibitors on viral entry prior to testing them in chronically infected patients.

## Conclusion

With most HCV clearance occurring in the first 90 minutes of transplantation, we believe that liver transplantation represents a prime opportunity to study the impact of HCV entry inhibitors.
